# Supplementary material for: Supervised topological data analysis for MALDI mass spectrometry imaging applications
Source: BMC Bioinformatics. 2023 Jul 10;24:279. doi: 10.1186/s12859-023-05402-0 (PMC10334630; doi:10.1186/s12859-023-05402-0)
Supplement: Supplementary file 1 — Additional file 1. The file contains the pseudo-code of the introduced algorithm and the proof of its complexity and correctness. [file 12859_2023_5402_MOESM1_ESM.pdf]

Additional file 1 — The introduced algorithm

This additional file provides the pseudo-code of the algorithm introduced in the paper *Supervised topological data analysis for MALDI mass spectrometry imaging applications.* Furthermore, it contains the proof of Theorem 1 of the paper.

Algorithm 1 Recursion start

```
Input: [f(x_0), ..., f(x_{q-1})]
Return: [(x̂, p(x̂)), ...]
maxima, minima, featurePairs ← ∅
for all x_j ∈ [x_0, x_{q-1}] do
    if x_j is maximum then
        maxima ← (x_j, f(x_j))
    else if x_j is minimum then
        minima ← (x_j, f(x_j))
SORT(maxima, f(x), >)
SORT(minima, f(x), <)
(x̂, f(x̂)) ← maxima.pop(0)
featurePairs ← (x̂, f(x̂) − minima[0][1])
RecursionStep(x_0, x̂, maxima.copy(), minima.copy(), featurePairs)
RecursionStep(x_n, x̂, maxima.copy(), minima.copy(), featurePairs)
return featurePairs
```

Algorithm 2 Recursion Step

```
Input: start, end, maxima, minima, featurePairs
for all (x_j, f(x_j)) ∈ maxima do
    if x_j ∉ [start, end) then
        maxima ← maxima\(x_j, f(x_j))
if |maxima| = 0 then
    return
(x̂, f(x̂)) ← maxima.pop(0)
RecursionStep(start, x̂, maxima.copy(), minima.copy(), featurePairs)
for all (x_j, f(x_j)) ∈ minima do
    if x_j ∉ (x̂, end) then
        minima ← minima\(x_j, f(x_j))
(x', f(x')) ← minima.pop(0)
featurePairs ← (x̂, f(x̂) − f(x'))
RecursionStep(x', x̂, maxima.copy(), minima.copy(), featurePairs)
RecursionStep(x', end, maxima.copy(), minima.copy(), featurePairs)
```

Proof of Theorem 1: The algorithm to calculate the reduced persistence transformation is divided into two parts: the recursion start (Algorithm 1) and the recursion step (Algorithm 2).

The *recursion start* (Algorithm 1) gets as input the list of all the intensity values for each m/z value, marked as $f(x_j)$. Let m be the number of maxima in this mass spectrum. In the beginning, empty lists are created for the maxima, the minima, and the results (called "featurePairs"). The latter list stores the x value, i.e., the position, as well as the persistence of each peak. Notice that each recursion step updates the list instead of returning results.

In the next step, all the minima and the maxima are stored in the corresponding lists in tuples of the form $(x, f(x))$. For this, the algorithm iterates through the list of the $f(x_j)$. If the value is larger than its neighbors, it is marked as maximum and stored in the corresponding list. Correspondingly, values that are smaller than their neighbors are marked as a minimum. This identification of extremal points can be made in linear run-time since the list is traversed just once, resulting in a complexity of $\sigma(q)$. The two lists *maxima* and *minima* are then sorted by their corresponding value $f(x_j)$ (the *minima* list inverted) with a complexity of $\sigma(m \cdot \log m)$.

For the largest feature, the global maximum, the persistence is defined to be the difference to the global minimum (see Equation (1) of the paper *Supervised topological data analysis for MALDI mass spectrometry imaging applications*). These values are the first elements of their corresponding lists. After calculating the persistence, the maximum is removed from the list, and the found feature $(x, p(x))$ is stored in the list *persistencePairs.* The *recursion step* is called afterwards with the intervals $[x_0, \hat{x})$ and $[x_{q-1}, \hat{x})$. Notice that the second interval is reversed. As input, the *recursion step* gets a copy of the two lists minima and maxima as well as the original list *featurePairs.* All these computations can be done in constant time, i.e., $\sigma(1)$. After the last *recursion step*, all the features are detected and stored in the *featurePairs* list and can be returned.

The input for the *recursion step* (Algorithm 2) consists of two indices, namely *start* and *end* (indicating the part of the data which is processed in the current recursion step, i.e., the positions of m/z values), a list of *maxima* and a list of *minima*, and the shared list of *featurePairs.* In the first step, the routine removes all the maxima not in the currently processed part of the data. There are at most m elements in the *maximum* list, so the complexity of this task is $\sigma(m)$. If the list is empty after the removing step, the *recursion step* reaches the end and can return. If not, it removes the first element $\hat{x}$ from the list. This is the most persistent feature (in terms of topology) in the processed part of the data. The elder rule (cf. [1]) states that the feature can only merge with a feature with a larger persistence, which is per construction at the index *end.* The corresponding minimum (cf. Equation (2) of the paper *Supervised topological data analysis for MALDI mass spectrometry imaging applications*) to $\hat{x}$ can only be in the interval $(\hat{x}, end)$ so the *minima* list can be filtered in a similar fashion to the *maxima* list with the same complexity. Since there are more possible features in the interval $(start, \hat{x})$, the *recursion step* is called once more for this interval.

The values $\hat{x}$ and the smallest minimum x' from the *minima* list generate a topological feature. This feature is updated in the original *featurePairs* list, and the recursion step can be repeated with the two intervals $(x', \hat{x})$ and (x', end).

The recursion step is processed at least once for each maximum with three extra calls after no more maxima are left, i.e., it runs at most $4 \times m$ times. Given the complexity of each step of $\sigma(m)$, the complexity of all the recursion steps together is $\sigma(m^2)$. This gives an overall complexity of the algorithm of

$$\sigma(q) + \sigma(m^2) + \sigma(m \cdot \log m) = \sigma(q) + \sigma(m^2).$$

For each maximum, there is a tuple stored which contains the information of the position and the persistence, resulting in an overall storage use of $2m$ elements.

The algorithm always terminates since, at each recursion step, one maximum is removed from the list of maxima —if it is not already empty. Likewise, each part of the input list is being processed. Since there is only a finite number of elements in the *maxima* list (i.e., m), the algorithm terminates after all are processed. Even more, the algorithm returns all the features with their persistence. Each maximum creates a feature, and all maxima are processed. They are paired with the correct minimum between themselves and a feature with a higher persistence according to the elder rule (see [1]). Hence, the algorithm always terminates and returns the correct solution in $\sigma(q)+\sigma(m^2)$ run-time. □

References

[1] Edelsbrunner, H., Harer, J.L.: Computational Topology: an Introduction. American Mathematical Society, Providence, USA (2010)
