# Supplementary material for: Supervised topological data analysis for MALDI mass spectrometry imaging applications
Source: BMC Bioinformatics. 2023 Jul 10;24:279. doi: 10.1186/s12859-023-05402-0 (PMC10334630; doi:10.1186/s12859-023-05402-0)
Supplement: Supplementary file 2 — Additional file 2. The file contains synthetic MALDI-images. Distinct types and levels of noise are added to the ground truth and displayed. Finally, the results of the denoising with the persistence transformation is depicted. [file 12859_2023_5402_MOESM2_ESM.pdf]

Additional file 2 — Additional simulation results

The additional file contains synthetic MALDI-images. Distinct types and levels of noise are added to the ground truth and displayed. Finally, the results of the denoising with the persistence transformation is depicted.

Gaussian Noise

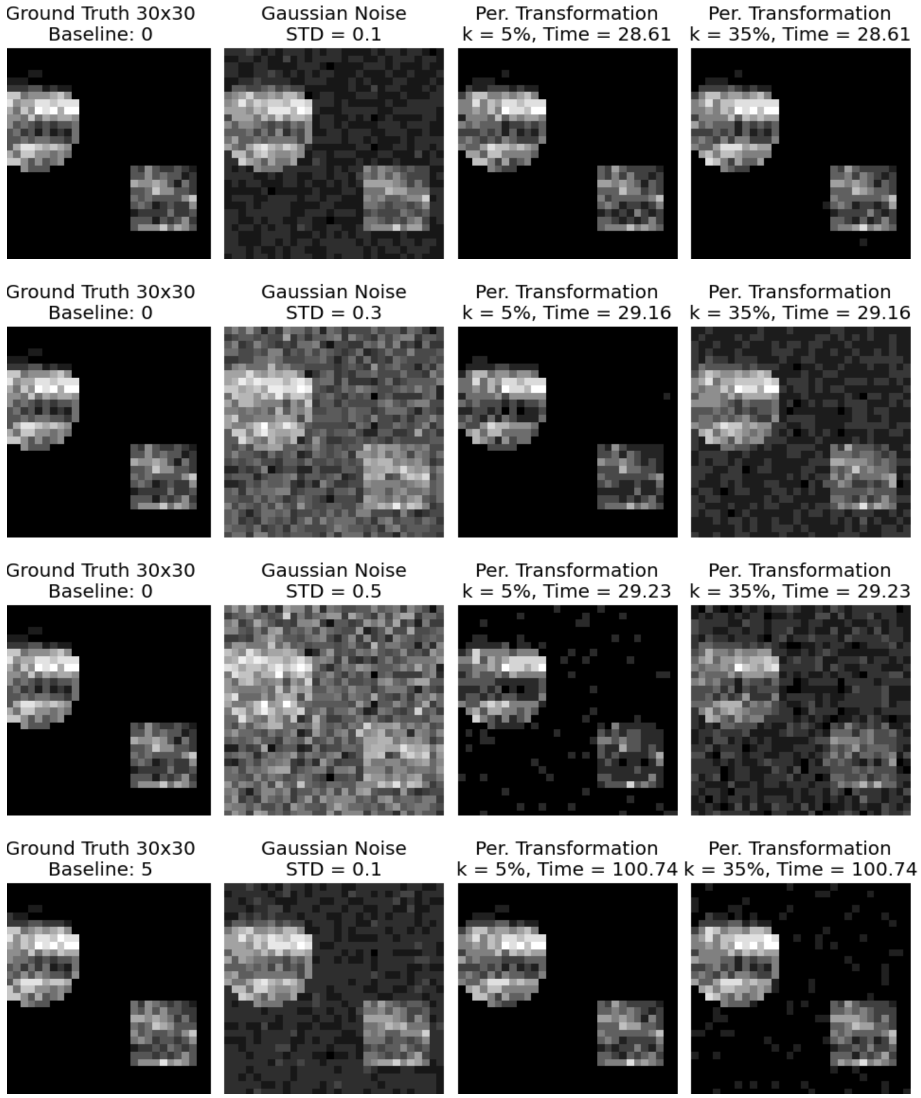

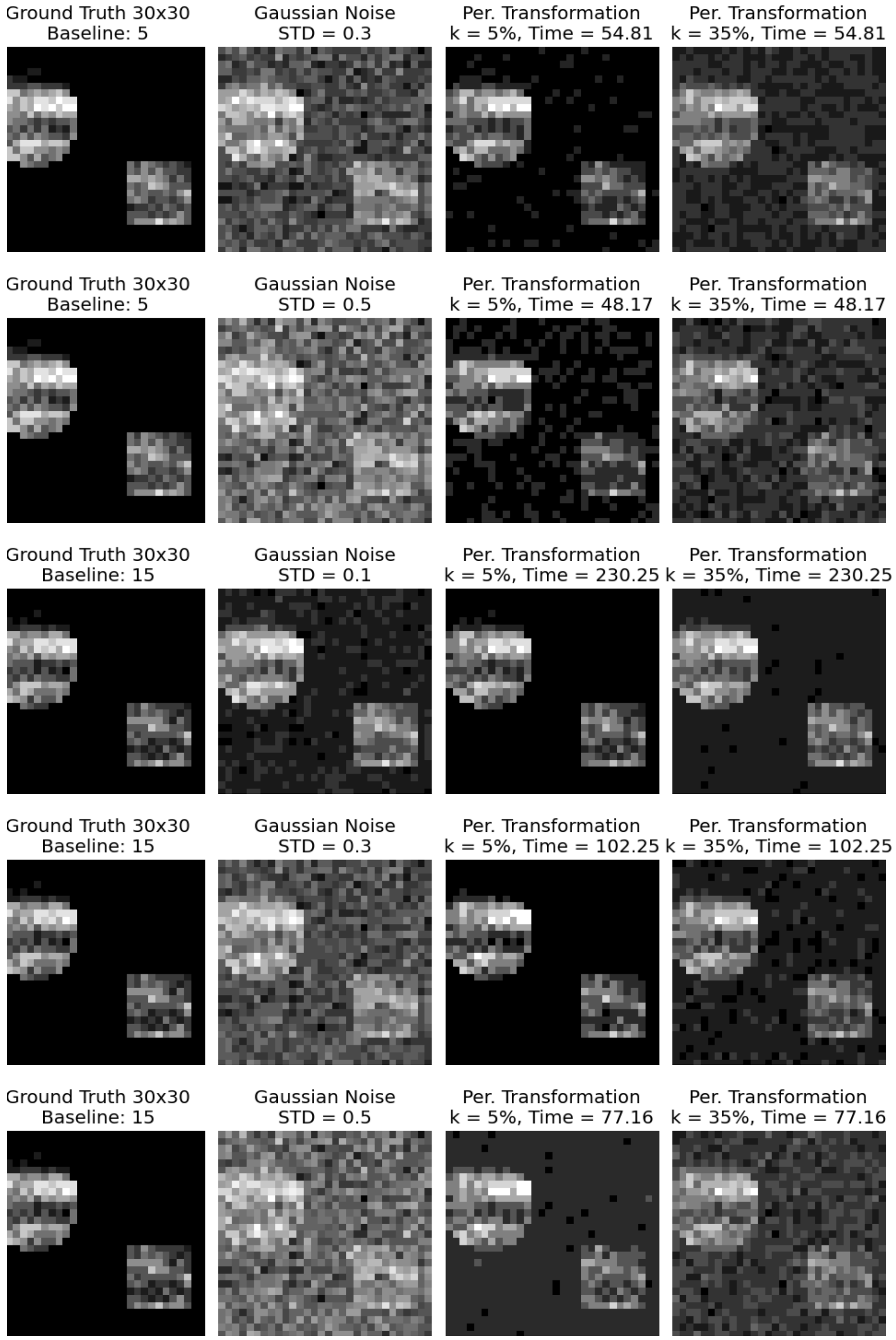
Ground Truth 30x30
Baseline: 5
Gaussian Noise
STD = 0.3
Per. Transformation
k = 5%, Time = 54.81
Per. Transformation
k = 35%, Time = 54.81
Ground Truth 30x30
Baseline: 5
Gaussian Noise
STD = 0.5
Per. Transformation
k = 5%, Time = 48.17
Per. Transformation
k = 35%, Time = 48.17
Ground Truth 30x30
Baseline: 15
Gaussian Noise
STD = 0.1
Per. Transformation
k = 5%, Time = 230.25
Per. Transformation
k = 35%, Time = 230.25
Ground Truth 30x30
Baseline: 15
Gaussian Noise
STD = 0.3
Per. Transformation
k = 5%, Time = 102.25
Per. Transformation
k = 35%, Time = 102.25
Ground Truth 30x30
Baseline: 15
Gaussian Noise
STD = 0.5
Per. Transformation
k = 5%, Time = 77.16
Per. Transformation
k = 35%, Time = 77.16

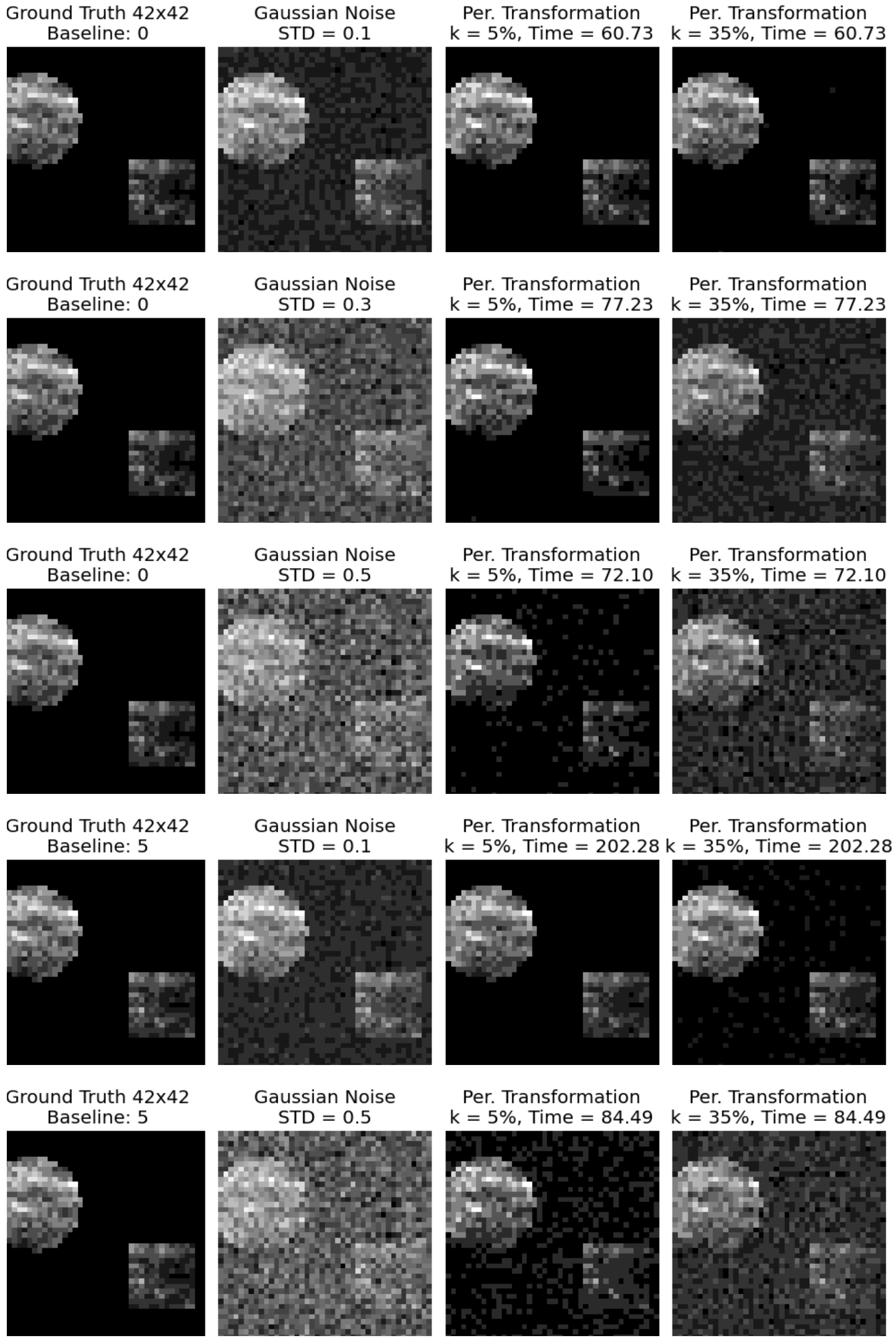
Ground Truth 42x42
Baseline: 0
Gaussian Noise
STD = 0.1
Per. Transformation
k = 5%, Time = 60.73
Per. Transformation
k = 35%, Time = 60.73
Ground Truth 42x42
Baseline: 0
Gaussian Noise
STD = 0.3
Per. Transformation
k = 5%, Time = 77.23
Per. Transformation
k = 35%, Time = 77.23
Ground Truth 42x42
Baseline: 0
Gaussian Noise
STD = 0.5
Per. Transformation
k = 5%, Time = 72.10
Per. Transformation
k = 35%, Time = 72.10
Ground Truth 42x42
Baseline: 5
Gaussian Noise
STD = 0.1
Per. Transformation
k = 5%, Time = 202.28
Per. Transformation
k = 35%, Time = 202.28
Ground Truth 42x42
Baseline: 5
Gaussian Noise
STD = 0.5
Per. Transformation
k = 5%, Time = 84.49
Per. Transformation
k = 35%, Time = 84.49

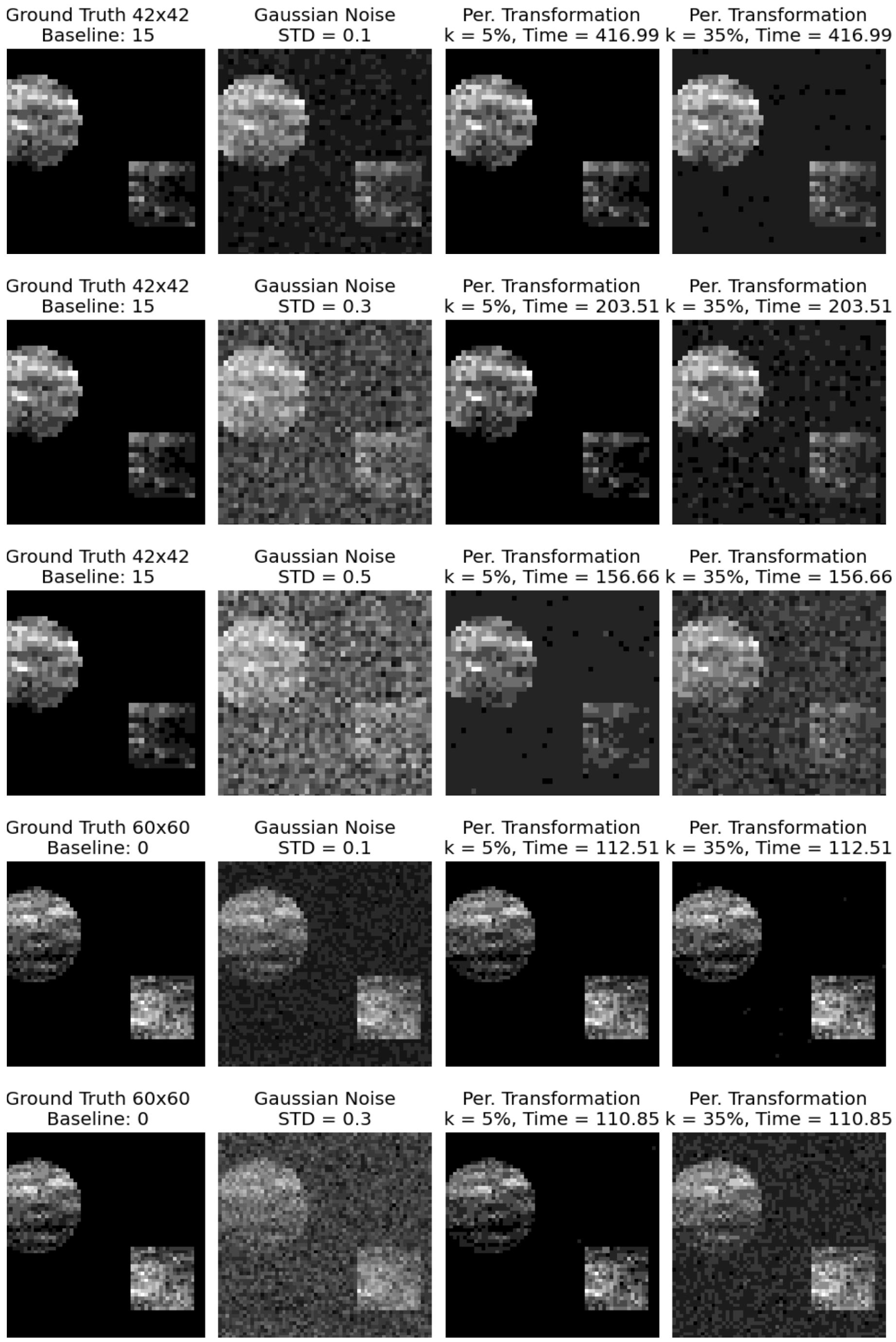
Ground Truth 42x42
Baseline: 15
Gaussian Noise
STD = 0.1
Per. Transformation
k = 5%, Time = 416.99
Per. Transformation
k = 35%, Time = 416.99
Ground Truth 42x42
Baseline: 15
Gaussian Noise
STD = 0.3
Per. Transformation
k = 5%, Time = 203.51
Per. Transformation
k = 35%, Time = 203.51
Ground Truth 42x42
Baseline: 15
Gaussian Noise
STD = 0.5
Per. Transformation
k = 5%, Time = 156.66
Per. Transformation
k = 35%, Time = 156.66
Ground Truth 60x60
Baseline: 0
Gaussian Noise
STD = 0.1
Per. Transformation
k = 5%, Time = 112.51
Per. Transformation
k = 35%, Time = 112.51
Ground Truth 60x60
Baseline: 0
Gaussian Noise
STD = 0.3
Per. Transformation
k = 5%, Time = 110.85
Per. Transformation
k = 35%, Time = 110.85

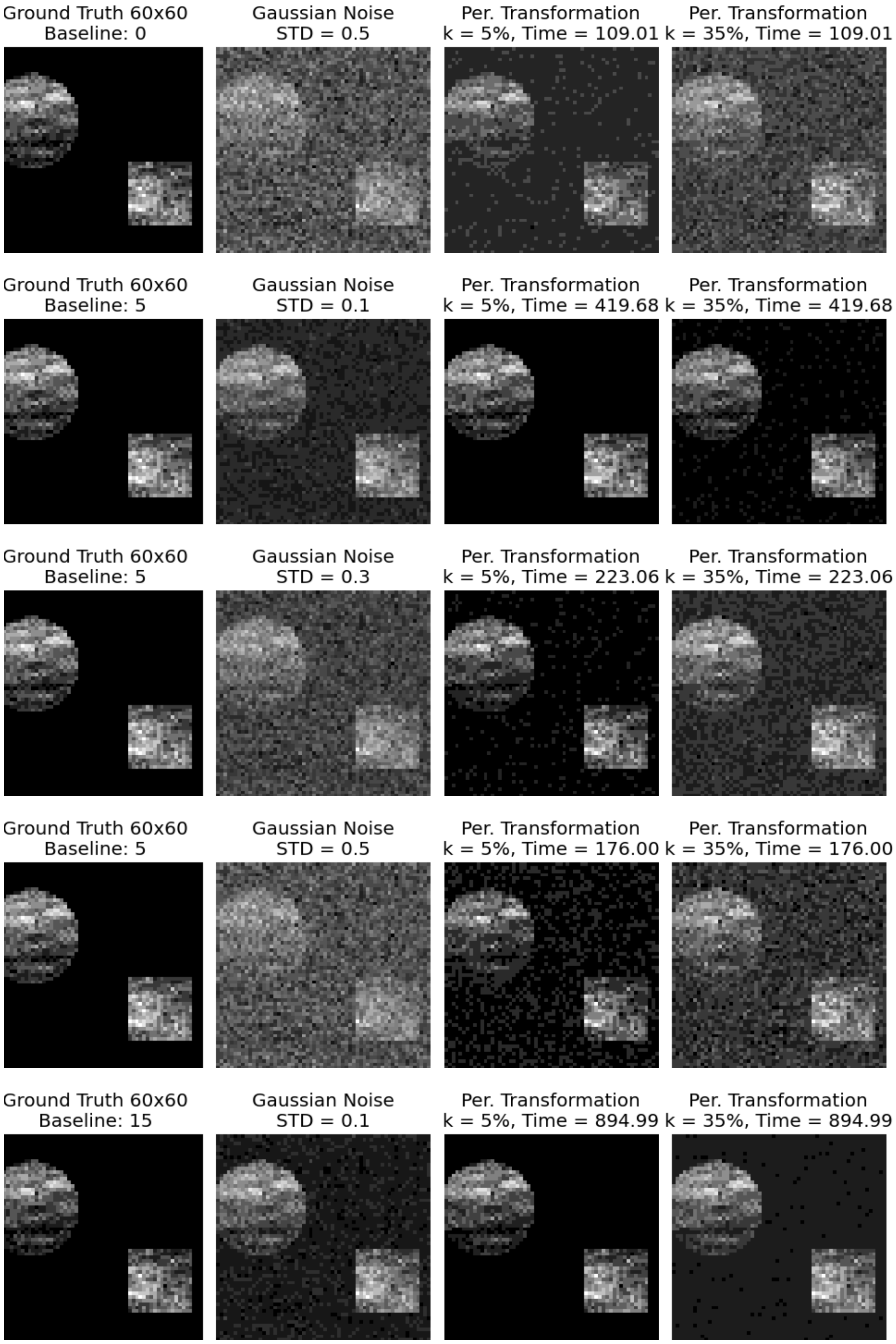
Ground Truth 60x60
Baseline: 0
Gaussian Noise
STD = 0.5
Per. Transformation
k = 5%, Time = 109.01
Per. Transformation
k = 35%, Time = 109.01
Ground Truth 60x60
Baseline: 5
Gaussian Noise
STD = 0.1
Per. Transformation
k = 5%, Time = 419.68
Per. Transformation
k = 35%, Time = 419.68
Ground Truth 60x60
Baseline: 5
Gaussian Noise
STD = 0.3
Per. Transformation
k = 5%, Time = 223.06
Per. Transformation
k = 35%, Time = 223.06
Ground Truth 60x60
Baseline: 5
Gaussian Noise
STD = 0.5
Per. Transformation
k = 5%, Time = 176.00
Per. Transformation
k = 35%, Time = 176.00
Ground Truth 60x60
Baseline: 15
Gaussian Noise
STD = 0.1
Per. Transformation
k = 5%, Time = 894.99
Per. Transformation
k = 35%, Time = 894.99

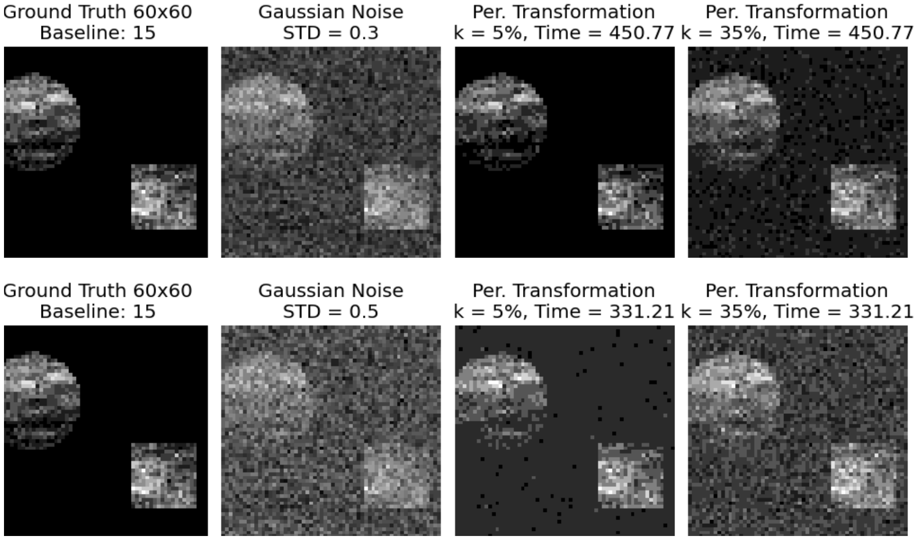

Poisson Noise

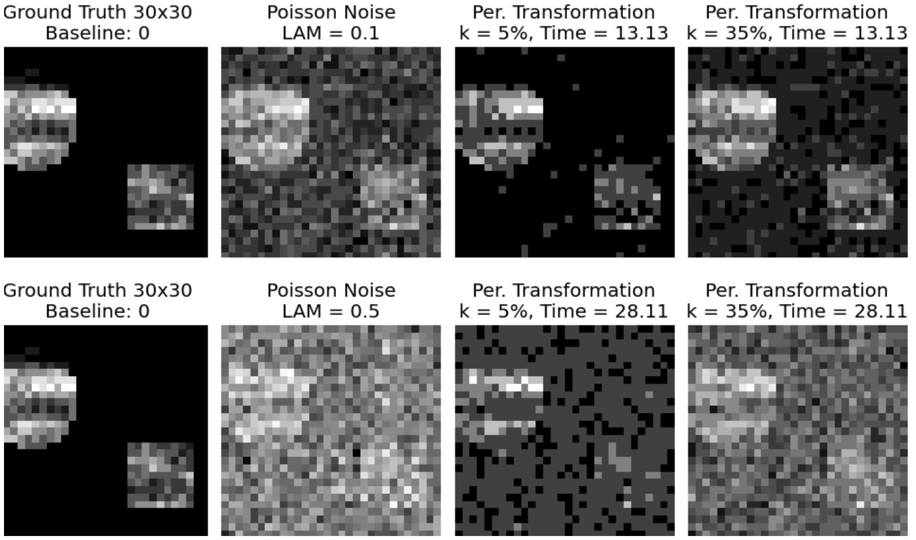

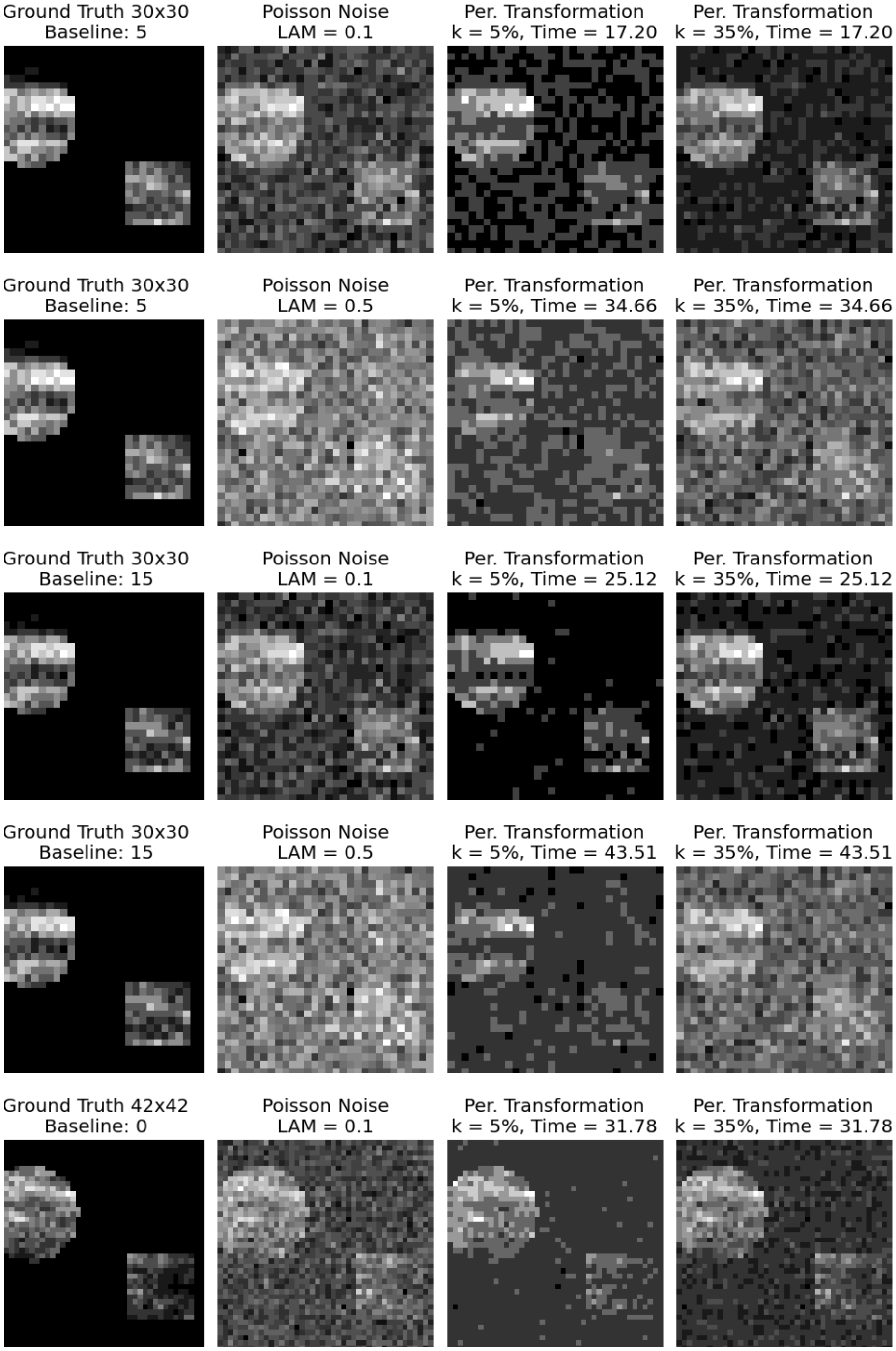
Ground Truth 30x30
Baseline: 5
Poisson Noise
LAM = 0.1
Per. Transformation
k = 5%, Time = 17.20
Per. Transformation
k = 35%, Time = 17.20
Ground Truth 30x30
Baseline: 5
Poisson Noise
LAM = 0.5
Per. Transformation
k = 5%, Time = 34.66
Per. Transformation
k = 35%, Time = 34.66
Ground Truth 30x30
Baseline: 15
Poisson Noise
LAM = 0.1
Per. Transformation
k = 5%, Time = 25.12
Per. Transformation
k = 35%, Time = 25.12
Ground Truth 30x30
Baseline: 15
Poisson Noise
LAM = 0.5
Per. Transformation
k = 5%, Time = 43.51
Per. Transformation
k = 35%, Time = 43.51
Ground Truth 42x42
Baseline: 0
Poisson Noise
LAM = 0.1
Per. Transformation
k = 5%, Time = 31.78
Per. Transformation
k = 35%, Time = 31.78

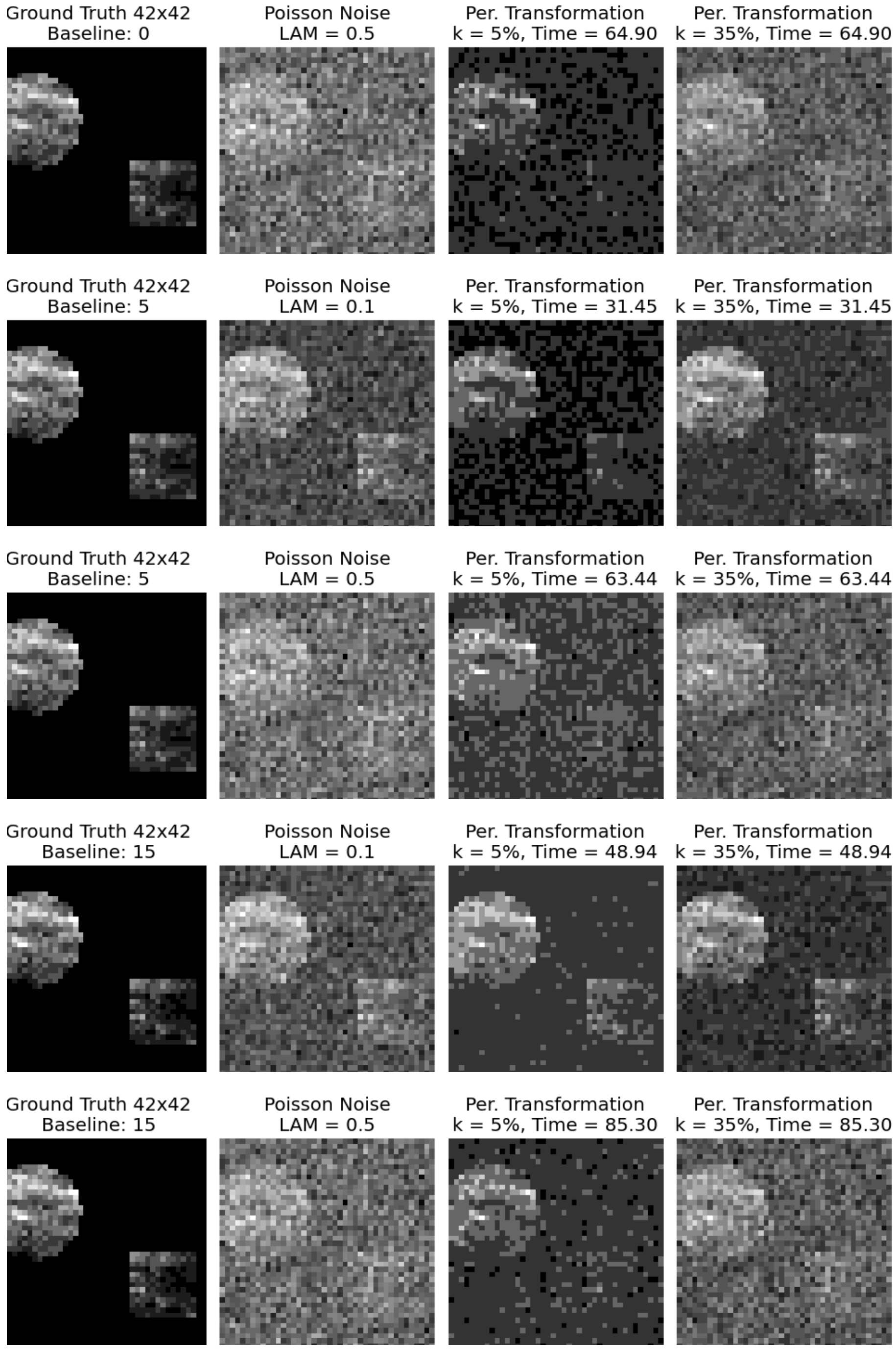
Ground Truth 42x42
Baseline: 0
Poisson Noise
LAM = 0.5
Per. Transformation
k = 5%, Time = 64.90
Per. Transformation
k = 35%, Time = 64.90
Ground Truth 42x42
Baseline: 5
Poisson Noise
LAM = 0.1
Per. Transformation
k = 5%, Time = 31.45
Per. Transformation
k = 35%, Time = 31.45
Ground Truth 42x42
Baseline: 5
Poisson Noise
LAM = 0.5
Per. Transformation
k = 5%, Time = 63.44
Per. Transformation
k = 35%, Time = 63.44
Ground Truth 42x42
Baseline: 15
Poisson Noise
LAM = 0.1
Per. Transformation
k = 5%, Time = 48.94
Per. Transformation
k = 35%, Time = 48.94
Ground Truth 42x42
Baseline: 15
Poisson Noise
LAM = 0.5
Per. Transformation
k = 5%, Time = 85.30
Per. Transformation
k = 35%, Time = 85.30

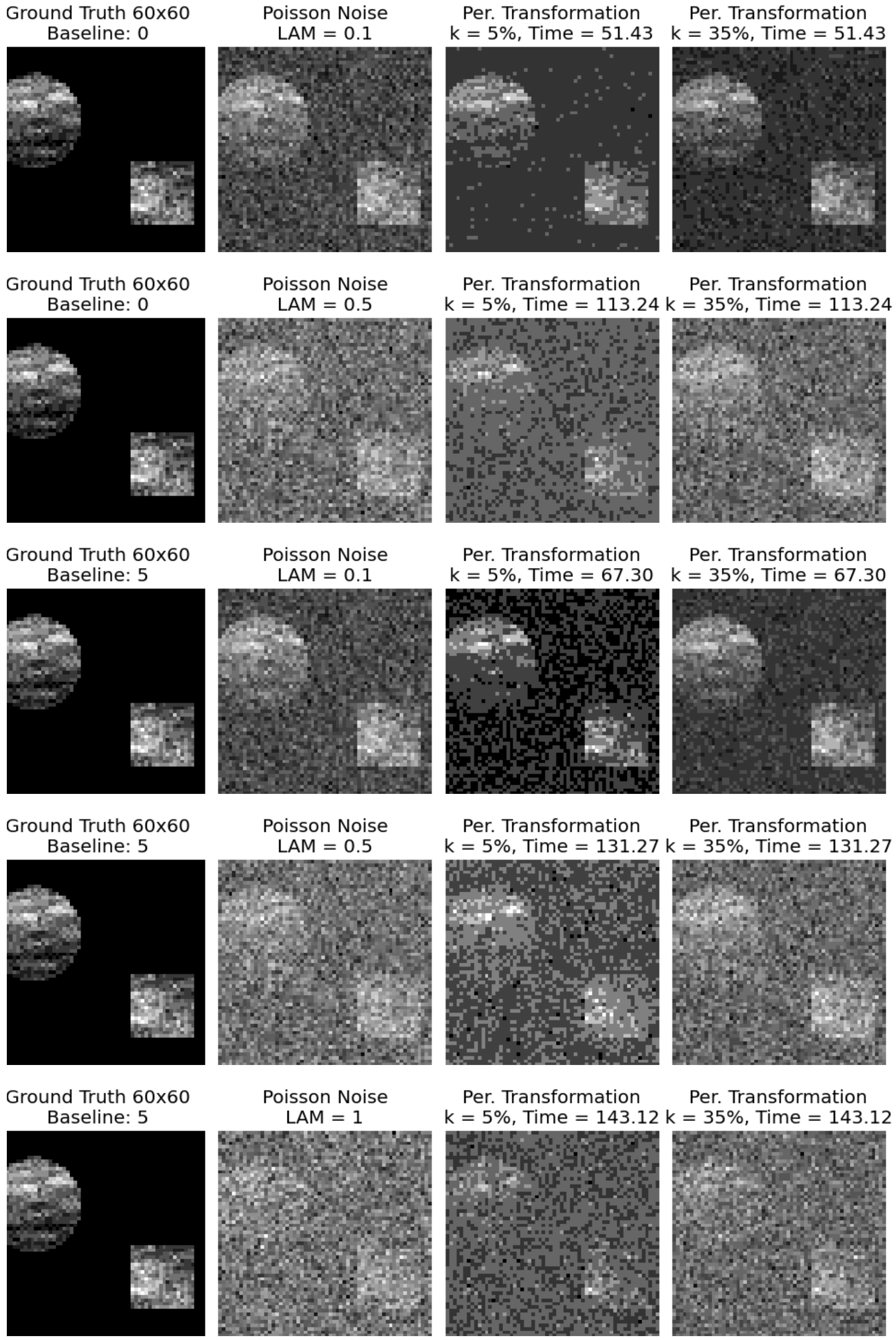
Ground Truth 60x60
Baseline: 0
Poisson Noise
LAM = 0.1
Per. Transformation
k = 5%, Time = 51.43
Per. Transformation
k = 35%, Time = 51.43
Ground Truth 60x60
Baseline: 0
Poisson Noise
LAM = 0.5
Per. Transformation
k = 5%, Time = 113.24
Per. Transformation
k = 35%, Time = 113.24
Ground Truth 60x60
Baseline: 5
Poisson Noise
LAM = 0.1
Per. Transformation
k = 5%, Time = 67.30
Per. Transformation
k = 35%, Time = 67.30
Ground Truth 60x60
Baseline: 5
Poisson Noise
LAM = 0.5
Per. Transformation
k = 5%, Time = 131.27
Per. Transformation
k = 35%, Time = 131.27
Ground Truth 60x60
Baseline: 5
Poisson Noise
LAM = 1
Per. Transformation
k = 5%, Time = 143.12
Per. Transformation
k = 35%, Time = 143.12
